# A Healed Intertrochanteric Femur Fracture, Shoulder, and Rib Fractures in an Ancient Nubian Female: An Osteoarchaeological Perspective

**DOI:** 10.1155/2024/8339694

**Published:** 2024-02-12

**Authors:** Randall T. Loder, Michele R. Buzon, Kaitlyn E. Sanders

**Affiliations:** ^1^Department of Orthopaedic Surgery, Indiana University School of Medicine, Indianapolis, IN, USA; ^2^Department of Anthropology, Purdue University, West Lafayette, IN, USA

## Abstract

This report is a case of a healed proximal intertrochanteric femur fracture nonunion in an ancient Nubian adult female, approximately 58 years old at the time of death, from the Tombos archaeological site in present day northern Sudan. Tombos was founded as an Egyptian colonial town during the New Kingdom Period (14001070 BC). The individual was radiocarbon dated to 1114-910 BC and also exhibited healed fractures of the left proximal humerus and ribs. There was shortening and mild atrophy of the right femur compared to the left; radiographs demonstrated a varus deformity of the proximal femur with associated retroversion. Bone density analysis revealed that the tissue mineral density z-score for this individual was −0.798, with the z-score for Tombos females 15–24 years old being 0.396, or a total difference of 1.194. This indicates that the individual was osteopenic but not osteoporotic prior to demise. This is an important case as it occurred approximately 3000 years ago and is the oldest known reported case of a healed intertrochanteric hip fracture in the archaeological literature. Archaeological cases of intertrochanteric hip fractures are rare, with none previously reported from the BC era. The timing of these multiple fractures is unknown, but all healed before the demise of the individual. Thus, there must have been considerable care afforded to such an individual to minimize the morbidities associated with nonoperative care of such a fracture. If all these fractures occurred at the same time due to a traumatic, accidental injury, the Modified Injury Severity Score (MISS) would be 25. Modern day trauma resuscitation and orthopaedic care gives an estimated mortality for such a MISS score of 28% for those <50 years old. It is likely that this individual's high socioeconomic status allowed for intensive nursing care which likely decreased the morality risk.

## 1. Introduction

This is a report of a well-healed, but malunited, proximal intertrochanteric right femur fracture in an ancient Nubian adult female who also sustained a proximal left humerus and two left rib fractures, which were also healed. This is an important case as it occurred approximately 3000 years ago and appears to be the oldest case of a healed intertrochanteric hip fracture in the archaeological literature. Today, intertrochanteric hip fractures typically occur in the elderly from simple falls [1, 2], while in younger adults (those whose femora have minimal osteopenia), they are due to higher energy events (motor vehicle crashes, falls from large height) [3]. Hip fractures in children are extremely rare [4]. In older individuals, nonoperative treatment mandates prolonged recumbency and often results in demise of the patient due to sepsis (pneumonia, urinary tract infections resulting in septicemia) and deep venous thrombosis (with secondary fatal pulmonary emboli) [5–7]. Even with modern medical management and urgent surgical internal fixation which allows for rapid mobilization, the mortality of these fractures is still high [8]. The 30-day mortality rate is 34% in those treated nonoperatively and 10% in those treated operatively [6]. The one-year mortality rate ranges from 12 to 37% [9]. In a recent study from Australia, the mortality rate for hip fracture patients was 3.5 times that of a noninjured hip fracture cohort matched for age and sex [10]. A recent study from the US found a one-year mortality rate of 20.2% in men and 17.3% in women [11]. This case is extremely unusual due to its archaeological age and that it demonstrated complete union before the demise of the individual. The additional left proximal humerus and rib fractures indicate further trauma and even polytrauma [12–15] if all the fractures occur simultaneously.

## 2. Materials and Methods

This individual was excavated in 2011 from the Tombos archaeological site in Sudan, located in what was ancient Nubia. The site was founded as an Egyptian colonial town during the New Kingdom period. The settlement and cemetery were used throughout the Napatan period (∼1400-650 BC) [16]. With permission from the National Corporation of Antiquities and Museums in Sudan, the remains were exported in 2011 to the Bioarchaeology Laboratory at Purdue University, West Lafayette, IN, USA.

The skeleton was from Unit 27 Burial 1 (calibrated [OxCal] radiocarbon dated to 1114-910 BC (95.4% NSF Arizona Laboratory AA95546 2841 ± 35 (2*σ*))), a tumulus tomb that dates to the late New Kingdom/early Napatan (Third Intermediate) period [17]. At the bottom of a traditional tomb shaft, a side chamber, extending the length of the shaft, was found to the north. Two burials of adult females were found, one (Burial 2) was partially articulated and in place (head to west—NSF Arizona AMS Laboratory AA95545: 2884 ± 36 (79.4% 1134-972 BC)). The other human remains (Burial 1) were scattered throughout the shaft and chamber. Wood and frass (decayed wood eaten by termites) remains suggest the use of a coffin for at least one of the burials. Frass bed pieces and bed feet also suggest the use of a funerary bed for at least one of the individuals. Several amulets were associated with the burials, with a cache found at the east end of the chamber. A large number of scattered faience and shell beads and smaller amulets were near where the head of the body would have lain, including a small Bes figurine, Pataikos, fish, and a very small scarab inscribed with a cobra and hieroglyphs. A cache of remarkable larger amulets excavated at the foot of the bed included two Isis figurines, an exceptionally fine quality Pataikos figurine, a large scarab with an unusual scene showing offering bearers carved on the base, along with multiple beads (including faience and shell). Metal remnants, likely handles to larger vessels, were also located near the foot of the bed.

### 2.1. Gross Specimens

**Figure 1:**
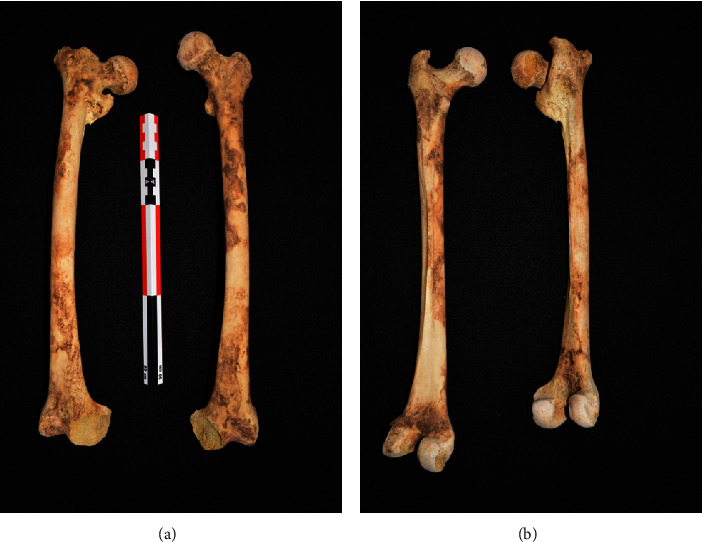
Frontal photographs of both femora. Note the significant shortening and the varus deformity of the proximal right femur (This is an original figure created by the authors of the manuscript). (a) Anteroposterior view. (b) Posteroanterior view.

The skeleton from Unit 27 Burial 1 was estimated to be a female based on skeletal features [18] and with a maximum likelihood age of 50.8 years [19]. The right femur length was 413 mm and the left was 439 mm. The midshaft mediolateral measurements were 23.38 mm on the right and 24.89 mm on the left; the midshaft anteroposterior measurements were 26.37 mm on the right and 26.71 mm on the left. This indicates atrophy of the right femur. The lengths of the tibiae were 364.00 mm on the left and 367.00 mm on the right. Based on the condition of the remainder of the skeleton, where the remaining bones were primarily intact (except for the fractures), the pathologic condition appears to have been caused by antemortem processes rather than taphonomic (changes after death) damage. There is visible shortening and mild atrophy of the right femur compared to the left (Figure 1).

The neck shaft angle of the abnormal right femur is 115° and the normal left femur is 130° (measurement techniques from Coon et al. [20] and Toogood et al. [21]). The femoral version is +17° (anteversion) on the left and −28° (retroversion) on the right. Normal ranges for the neck shaft angle and femoral anteversion are 129° ± 6° and 10° ± 9°, respectively [21].

### 2.2. Radiographic Analysis

**Figure 2:**
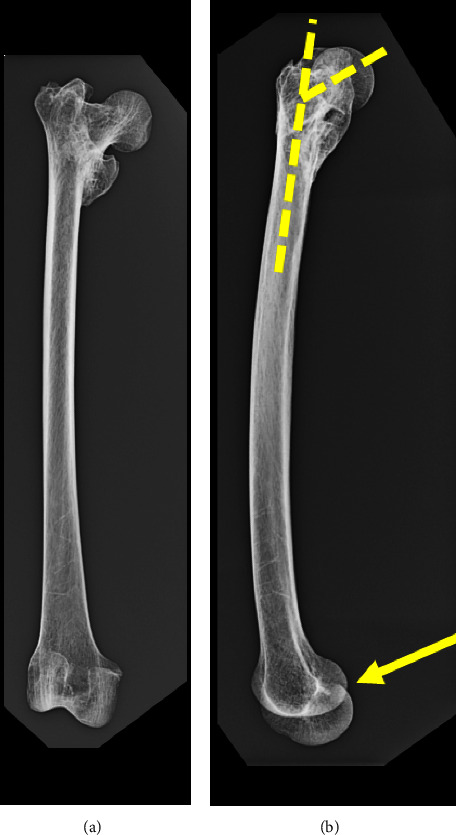
Radiographs of the entire femur (This is an original figure created by the authors of the manuscript). (a) Anteroposterior projection. (b) Lateral projection. Note that with the femoral condyles rotationally the same (solid yellow arrow), there is retroversion of the femoral head/neck relative to the shaft (hatched lines).

Conventional anteroposterior and lateral radiographs of the entire femur document a varus deformity of the proximal femur with associated retroversion (Figure 2).

**Figure 3:**
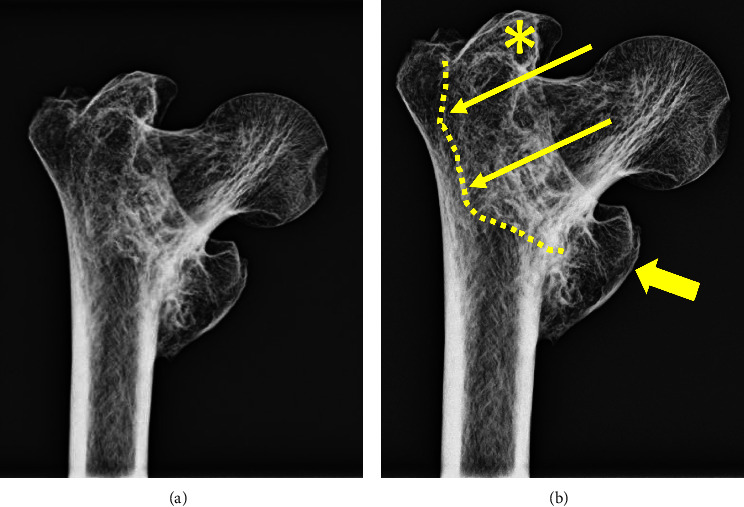
Anteroposterior radiograph of the hip (This is an original figure created by the authors of the manuscript). (a) Without markings. (b) Markings that demonstrate the greater trochanter abducted and medially displaced (yellow asterisk) along with exuberant callus in the region of the lesser trochanter (solid yellow arrow). The faint appearance of an old fracture line can be seen (hatched line and thin yellow arrows).

**Figure 4:**
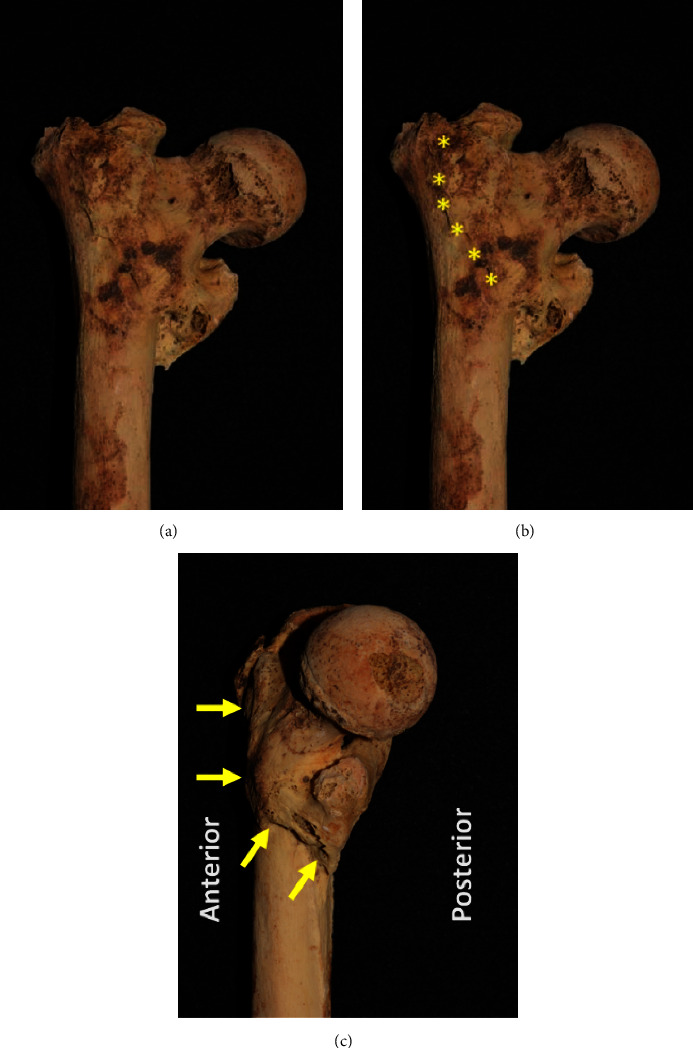
Photographs of the physical specimen focused on the proximal femur (This is an original figure created by the authors of the manuscript). (a) The same position as in the radiograph. (b) The major fracture line denoted by yellow asterisks. (c) A view from directly medial to lateral. Note the clearly seen, well-healed fracture (yellow arrows) exiting just inferior to the lesser trochanter and the retroversion of the proximal femur superior to the fracture line.

**Figure 5:**
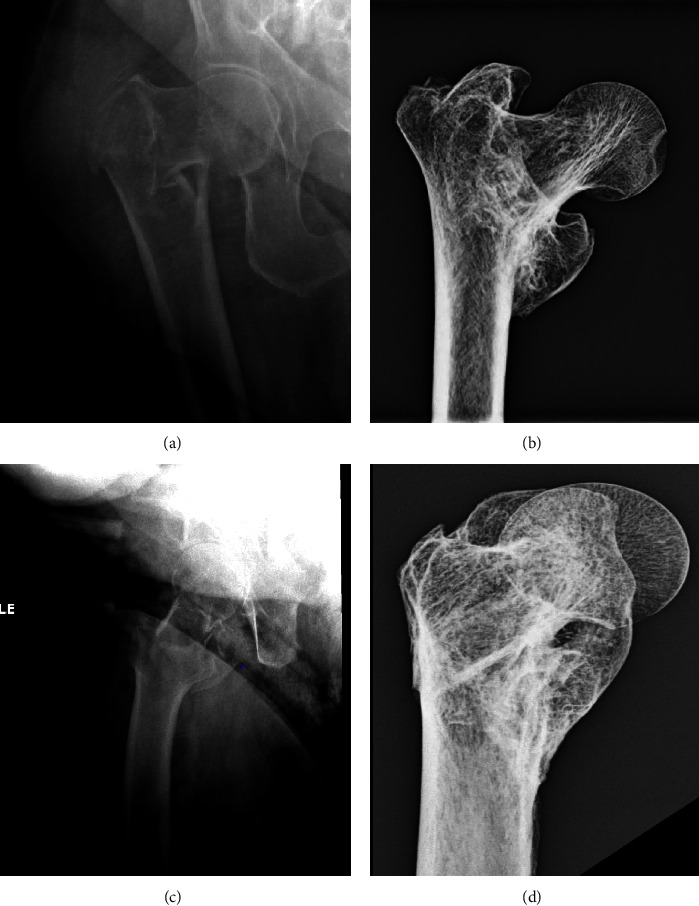
Comparison of the Tombos specimen to a present-day fracture in a present-day patient (This is an original figure created by the authors of the manuscript). (a) The AP radiographs of the present-day individual; note the marked adduction of the distal femoral shaft. (b) The AP radiograph of the healed fracture in the Tombos individual. (c) The lateral radiograph of the present-day individual; note the retroversion of the femoral neck and head. (d) The lateral radiograph of the healed fracture in the Tombos individual.

The well-organized, healed callus and varus malunion are seen in dedicated radiographs of the right hip (Figure 3) and the gross specimen (Figure 4). The striking resemblance of this malunion is compared to that of a present-day individual with a similar intertrochanteric femur fracture (Figure 5).

**Figure 6:**
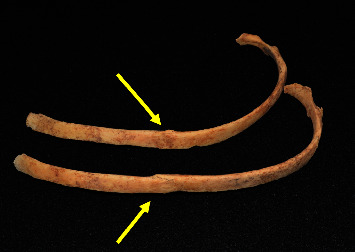
Photograph of the healed rib fractures (yellow arrows) (This is an original figure created by the authors of the manuscript).

**Figure 7:**
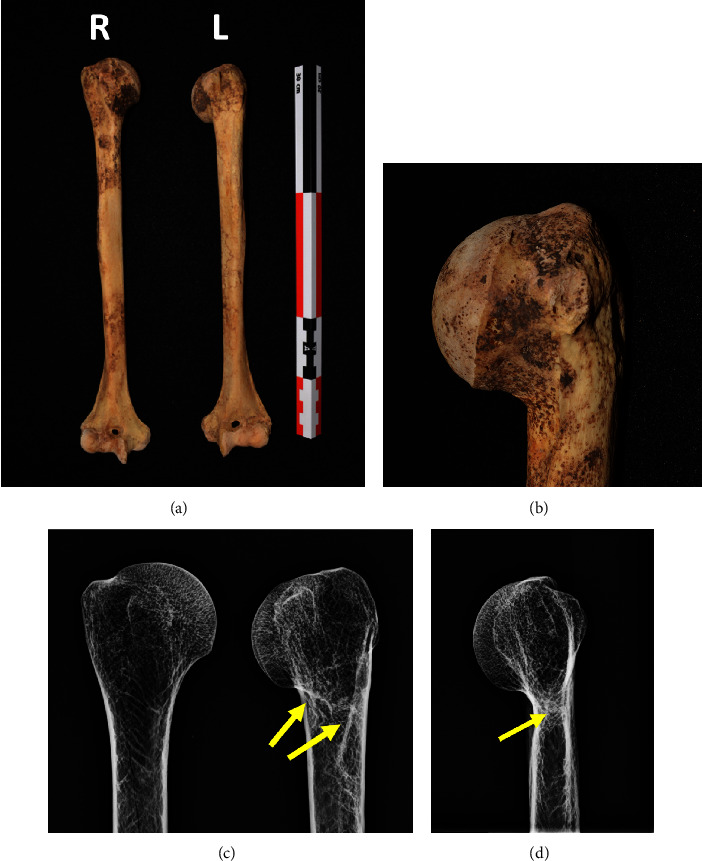
A well-healed fracture of the left humerus surgical neck (This is an original figure created by the authors of the manuscript). (a) An anteroposterior photograph of both the right and left humerii. Note the shorter left humerus; the left humerus was 309 mm in length and the right one was 323 mm in length. (b) A close-up photograph of the left proximal humerus demonstrating a varus neck shaft position with inferior displacement of the humeral head. (c) An anteroposterior radiograph of both proximal humerii. Note the internal callus of the left humerus denoting a well-healed longstanding fracture (yellow arrow). (d) A lateral radiograph of the left proximal humerus demonstrating the internal callus (yellow arrow) and distorted surgical neck. Malunions such as this are common in fractures of the humeral neck treated nonoperatively [22].

In addition to the major femur fracture, there were also 2 well-healed left rib fractures (Figure 6) and a well-healed fracture of the left surgical humeral neck (Figure 7). Aside from these fractures, the only other abnormalities noted after a thorough visual inspection of the skeleton were some mild degenerative changes of the cervical and lumbar spine as well as five tooth abscesses, conditions that are common for individuals of this age in the Tombos individuals.

### 2.3. Bone Density Analyses

Bone density analyses have been completed on 84 of the individuals from the Tombos collection [23] using two different methods. The first was the trabecular tissue mineral density (TMD) using the fourth lumbar vertebrae (or third if the fourth was not available). Trabecular tissue mineral density (TMD) was used as the marker for osteopenia/osteoporosis; bone mineral density (BMD) is used in clinical studies and assesses the overall density of cortical bone, trabecular bone, and intertrabecular spaces (soft tissue). Tissue mineral density (TMD) refers specifically to the density of the trabecular bone and does not include cortical bone or soft tissue estimates. Due to the nature of the archaeological skeletal material, namely, the absence of soft tissue, the density values used in this study reflect the trabecular tissue mineral density rather than the clinical BMD. The raw TMD values for this individual as well as the reference sample were converted to standardized z-scores based on the population mean (Addendum 1). For this particular individual, the TMD z-score was −0.798, while the TMD z-score for Tombos females who are 15–24 years old was 0.396 or a total difference of 1.194.

The second method was the 2^nd^ metacarpal cortical index (CI) as defined by Ives and Brickley [24]. The measurements were the length (L), total width at midpoint (TW), and medullary width at midpoint (MW). With these measurements, CI = ((TW − MW)/TW) *∗* 100. The average CI for Tombos females aged 15–24 years was 57.78; the CI for this individual was 33.94 (58.7% of the young female group).

## 3. Discussion

While the femur finding is most likely a healed, malunited intertrochanteric femur fracture, other possibilities should be considered. Following this differential diagnosis, the proximal humerus and rib fractures will be addressed.

### 3.1. Differential Diagnosis of the Femur

#### 3.1.1. Vascular

Coxa vara is a common sequelae of Legg–Calve–Perthes' disease [25, 26]. However, there is no exuberant bone formation in the lesser trochanteric area as in this specimen, and there is usually some mild femoral head deformity, not seen here as the femoral head is very spherical.

#### 3.1.2. Neoplastic

Osteochondroma lesions of the proximal femur are well described but are usually in patients with multiple hereditary exostoses [27–29] but are typically bilateral along with many other sites of involvement aside from the proximal femur; most importantly, the femoral deformity is valgus rather than varus. In this case, the lesion is isolated, unilateral, with a varus deformity, making such a diagnosis unlikely.

#### 3.1.3. Developmental

Slipped capital femoral epiphysis can result in a mild varus deformity of the proximal femur with retroversion. However, the pathologic deformity is at the junction of the proximal femoral metaphysis and epiphysis, which is normal in this case [30]. Developmental dysplasia of the hip (DDH, earlier known as congenital dislocation of the hip) [31–33], ranging from simple acetabular dysplasia to frank dislocation, can result in apparent foreshortening of the lower extremity. However, it is not likely for this case as even severe untreated DDH does not result in a varus deformity with exuberant bone in the lesser trochanteric region.

#### 3.1.4. Congenital

Congenital coxa vara is a rare but well-described entity typically associated with retroversion and femoral shortening [34, 35]. However, the greater trochanter is normally positioned (i.e., not lateral/medial) relative to the femoral shaft, which is present in this case. Aside from multiple proximal femoral osteotomies to correct this varus deformity, there is no exuberant bone in the lesser trochanteric area [34].

#### 3.1.5. Infectious

Osteomyelitis often results in periosteal new bone [36–38]. However, in the absence of a pathologic fracture, a varus deformity would not occur. Also, after the resolution of the acute infectious episode, there will typically be a sequestrum inside the femoral region, which is not seen here. Finally, the new periosteal bone formation is seen circumferentially, unlike the exuberant formation seen only in the lesser trochanteric area in this case.

Thus, a healed intertrochanteric proximal femur fracture is the most probable explanation of this finding. Intertrochanteric hip fractures are typically classified as 2-part, 3-part, or 4-part patterns [39] and stable or unstable [5]. This fracture was most likely an unstable 2-part or 3-part fracture due to the femoral retroversion, shortening, and varus deformity of the proximal femur. This position in untreated fractures is due to the musculotendinous attachments on the respective fragments creating the deformity [1]. The gluteus medius and short external rotator abduct and externally rotate the greater trochanter, while the adductors and hamstrings pull the femoral shaft into an adducted (varus) position.

A similar protocol could be followed for the rib and proximal humerus abnormalities, but the same arguments would apply, and both the proximal humerus and rib anomalies are most likely fractures.

### 3.2. Bone Mineral Density

This individual had osteopenia relative to her own Tombos population. The diagnostic criteria for osteopenia, as defined by WHO, is a BMD of between 1 and 2.5 standard deviations below the average value for young, healthy adults from the reference group (in this case, females) [40]. The overall difference based on the standardized TMD was −1.194 compared to Tombos females, 15–24 years old. This is −1.88 standard deviations below the mean of young females from this population. The 2^nd^ metacarpal cortical index was 33.94 or 58.7% of the same female group, 15–24 years old. These comparisons reveal signs of cortical and trabecular bone loss. Compared to females within a similar age/sex group, this individual has high trabecular separation and mean intercept length, which may have contributed to bone fragility [23]. Using today's standards, the individual was clearly osteopenic as well using the cortical index. Patel et al. [41] have shown that a CI of 41.5 or less is diagnostic of osteopenia and directly correlates with the hip DEXA t-score. Haara et al. [42] found that those with a metacarpal cortical index <0.44 had 6.3 times relative risk of sustaining a hip fracture compared with those with an index >0.70. Of the 357 individuals with a cortical index >0.70, one developed a hip fracture (0.3%); of the 356 individuals with an index <0.44, 42 developed a hip fracture (11.8%). Thus, this Tombos individual, with a cortical index of 33.9, was at a high risk of developing a hip fracture.

### 3.3. Archaeological Records

Archaeological cases of intertrochanteric hip fractures are rare, and those that progressed to union before death are even rarer. We were unable to locate any published archaeological case of an intertrochanteric hip fracture in the BC era. In a review of 52 mummies from ancient Egypt searching for the orthopaedic pathology [43], not a single hip (intertrochanteric or femoral neck) fracture was noted. Dequeker et al. [44] described a hip fracture in a XIIth dynasty (1990-1786 BC) female from Lisht, Upper Egypt, but it was of the femoral neck.

There are many more cases of hip fractures in CE archaeological specimens. A united femoral neck fracture with varus deformity was described in a Roman male from an Erculam cemetery [45]. This malunion resulted in a limb length discrepancy, but the magnitude was not stated. This fracture was believed to be a fragility fracture, although no bone density measurements were obtained. Lovell [45] believed that the coxa vara and length discrepancy was enough to require the individual to weight bear on his toes during gait to compensate for the limb shortening. Upon critical review of the photographs in the manuscript, it would appear that the fracture was at the base of the neck; however, no radiographs were shown to help differentiate. This is important as displaced fractures of the midfemoral neck have a high rate of both avascular necrosis as well as nonunion, neither of which occurred in Lovell's case; thus, from an orthopaedic perspective, the fracture was likely basicervical. Lovell [45] noted degenerative changes at the 1^st^ metatarsophalangeal joint, attributed to walking over time with a significant leg length discrepancy, and argued that the individual described compensated for the shortened lower extremity by a functional lengthening by bearing weight on the toes when walking, rather than striking the heel first [46] and then pushing off with the toes. This prolonged pushing off with the toes led to the development of degenerative disease in the first metatarsophalangeal joint as well as the knee, indicating that the injury occurred years before the man's death. With this in mind, we specifically reviewed this individual, and no degenerative changes in either the right foot or knee were noted. Thus, this Tombos individual likely sustained this hip fracture in the recent past but with enough time to allow for total union but not long enough ago to develop degenerative changes in the foot or knee.

The next cases are from the 2^nd^ millennium CE. McKenzie et al. [47] have described a traumatic obturator fracture dislocation of the left hip in a male, aged 35–50 years at the time of death, from the Late Medieval Gaelic burial ground of Ballyhanna, Co. Donegal (c. 1200–c. 1600 CE). While not a true hip fracture, the survival of such an individual would have necessitated prolonged and meticulous nursing care in the early phases of the injury, and likely significant care after healing, as this injury markedly inhibits mobility and is very disabling. Such prolonged bed rest would typically result in an early demise due to all the same complications that occur with bed rest treatment of adult hip fractures, which did not occur in this case.

The postmedieval period supplies more cases. Ives et al. [48] described 15 hip fractures out of 1,597 individual skeletons recruited from eight postmedieval urban sites in England from the eighteenth and nineteenth centuries. Three (20%) of these fractures involved the intertrochanteric region. Of these three, one had healed and two were healing at the time of death. They noted that intracapsular femoral neck fractures were less likely to have rapid death with some survivors surviving after a longer period after the fracture, whereas subcapital fractures were associated with an earlier demise. However, the distinction between subcapital and intertrochanteric fractures is not described in detail. They found that the survival rate was greater in males than in females.

The Portuguese archaeological record discusses 6 hip fractures [49]. The first and oldest case was from 3000 to 2500 BC female with a femoral neck (intracapsular) fracture. The remaining 5 cases were CE dated. The 2^nd^ case is an intertrochanteric fracture in a male >50 years old with nonunion from the Paradela churchyard (12^th^–19^th^ centuries). The 3^rd^ case is from the Convent of São Francisco, Santarém (14^th^–17^th^ centuries), of a female >50 years old with a healed intertrochanteric fracture. The 4^th^ case is from the Church of Sãno Julião, Constância (14^th^–19^th^ centuries), of a female >50 years old with a healed valgus impaction fracture of the femoral neck. The 5^th^ case is from the Juncal necropolis (16^th^–20^th^ centuries) of a male with a probable femoral neck fracture, although the possibility of a slipped capital femoral epiphysis was raised. As no photographs or radiographs are shown, it is difficult to comment further. The 6^th^ case is from the Church of Nossa Senhora da Conceiação, Seixal (18^th^-19^th^ centuries), of a male >50 years old with an unhealed comminuted fracture of the right femur. Another case [50] was described from the Church of Nossa Senhora da Anunciada (Sertúbal, Portugal) (16^th^–19^th^ centuries) of an extracapsular femur fracture, which from an orthopaedic perspective was a healed nondisplaced intertrochanteric femur fracture.

There are two studies from recent skeletal collections. The first involves the Robert J. Terry collection [51], which originated from St. Louis, Missouri, from 1823 to 1947 [52]. Mant et al. [51] reviewed 10 individuals from the Terry collection with documented hip fractures; 70% were intertrochanteric. Of these 10 cases, 8 were severely comminuted and died within four months of sustaining the fracture. The second study is from the Hamann–Todd collection [53]. They reviewed 938 skeletons and found that 34 had sustained proximal femur fractures, 22 were extracapsular and 12 were intracapsular. Union was seen in 23% of the extracapsular and 87% of the intracapsular fractures at the time of death. As the present case is an extracapsular fracture, the fact that this person survived is remarkable.

Intertrochanteric fractures of the femur, even with urgent surgical fixation and mobilization, have significant mortality [5, 6, 8, 10, 11], especially in the elderly. Evans [5] in his seminal paper noted that “very old patients who sustain this injury tolerate pain and immobility badly; their mental state is often precarious, and they are quick to develop bedsores or pulmonary complications.” This is supported by the recently described cause of death of Emperor Charles IV, King of Bohemia, which was deciphered to be pneumonia secondary to being recently bedridden after sustaining a left femoral neck fracture, with the specimen documenting no signs of healing [7].

Contemporary orthopaedic treatment of intertrochanteric fractures is surgical internal fixation with one of several devices along with a reduction (anatomically positioning the fracture, typically by closed means on a fracture table with longitudinal traction, internal rotation, and slight abduction of the distal fragment). This fixation allows for rapid mobilization of the patient as well as realigning the femur to a reasonably normal neck shaft angle and version. Fracture union then occurs in a normal time frame, but the patient is mobilized, upright, and ambulatory before complete union due to the internal fixation.

The time to union of an intertrochanteric fracture treated with bed rest (in those patients that actually survive) is an average of 15 weeks before mobilization was allowed [5]. This case documented total union with remodeling of the bony trabeculae and smoothing of the callus; thus, the time of demise of this individual is far greater than 15 weeks from the time of the fracture. The exact time, of course, is not known.

Regardless of the exact time when the fracture occurred before the demise of the patient, several interesting points should be noted. There must have been considerable care afforded to such an individual to minimize the morbidities associated with nonoperative care of such a fracture. This has been recently noted in a medieval case of an obturator fracture dislocation of the hip [47] as well as other fractures [54] from the medieval period. Dittmar et al. [54] described three individuals from medieval Cambridge, England, with multiple fractures. One interesting case involves a female 45–65 years old at the time of death with several fractures. There were healed fractures of the right femoral neck, right forearm, several ribs, the fourth lumbar vertebra, and right patella. In modern terminology, this would be polytrauma.

### 3.4. The Possibility and Consequences of Polytrauma

Polytrauma is defined as multiple injuries occurring at the same time [12–15]. This woman clearly sustained multiple fractures, but of course, it is unknown if they were all simultaneous. However, all four fractures demonstrated complete union before death. Thus, these fractures must have occurred earlier in life with enough time to allow for total union, but not markedly earlier, as no degenerative changes in the foot and knee were noted, which likely would have been present as described by Lovell [45]. The intertrochanteric femur fracture was clearly relatively recent due to the fact that no degenerative changes in the foot and knee were noted [45], and as the individual was estimated to be 51 years old at the time of death, it would clearly be after the time of remodeling seen with pediatric fractures, especially with proximal humerus fractures [55, 56]. Thus, the argument that the hip and shoulder fractures occurred in childhood/adolescence is mute as it is well known that there is considerable bony remodeling after such fractures [57] which would result in remodeling that would be visualized on radiographs.

Assuming that all these fractures occurred at the same time due to a traumatic, accidental event, a severity score with a probability of mortality can be calculated. Severity scoring of the multiply injured patient [13–15] using the Modified Injury Severity Score (MISS) is well known and is used in the adult trauma literature. These scoring systems can predict the mortality of a patient with multiple injuries by age categories (remembering that this involves modern-day management of the injured patient, which was not in existence at the time of this individual). The MISS uses the Abbreviated Injury Scale (AIS) for five different body areas: the face and neck, chest, abdomen, musculoskeletal, and neural. The AIS categorizes the injury severity within each body area. This individual sustained injuries to at least two different areas: chest (rib fracture) and musculoskeletal (femur and humerus fracture). The MISS calculates the score on the severity of the injury within each body area. The MISS is calculated by adding the sum of the squares of the three most severely injured AIS body areas. In this particular case, there are at least two known body areas: the chest and the musculoskeletal areas. The AIS score for 2 rib fractures is 3, and the AIS score for 2 long bone fractures is 4; thus, the MISS for this individual would at least be 3^2^ + 4^2^ or 25. Studies using modern-day trauma resuscitation and orthopaedic care give an estimated mortality for a MISS score of 25 of 10% in the 1974 study [13] (which was not age adjusted) and 27.7% in those <50 years of age in a 1988 study accounting for age adjustment [15]. Thus, the fact that this individual survived multiple fractures that healed before demise is remarkable, as even with today's trauma management regimes, the mortality is ∼28%. This percentage would much likely be higher in the time for this individual from ∼1000 BC.

The survival of such an injury would require significant supportive care around the clock, as the person would not be able to walk for several months and eating/drinking and toileting would likely need prolonged assistance for a prolonged time as well. As there were amulets, figurines, beads, and vessels in this burial, the individual was likely from a high socioeconomic position having the resources to provide such intense care. This is similar to the case described earlier by McKenzie et al. [47]. As there were no degenerative changes in this individual in the foot/ankle, indicating that the fractures occurred relatively recently, they also thus occurred when the individual was osteopenic as seen from the bone density analyses.

In conclusion, this case documents a well-healed, but malunited, proximal intertrochanteric right femur fracture in an ancient Nubian adult female from approximately 3000 years ago who also sustained a proximal left humerus and two left rib fractures which also healed. This is an important case for several reasons: (1) it appears to be the oldest case of a healed intertrochanteric hip fracture in the archaeological literature, (2) the patient was osteopenic, typical of today's patient with an intertrochanteric hip fracture, and (3) additionally, healed rib and proximal humerus fractures were also present, likely indicating polytrauma. The fact that this individual survived multiple fractures with complete union before death is remarkable, as even with present-day trauma management, the mortality is at least 25%. It is likely that this individual's high socioeconomic status allowed for intensive nursing care which likely decreased the morality risk.

### 3.5. Addendum 1

The 3D vertebral scans were analyzed using Analyze 12.0 software (BIR, Mayo Clinic, 1999–2014). Scans were imported and manually edited to include only the middle one-third section of the trabecular bone of the vertebral body, excluding the cortical bone, effectively manually determining the region of interest (ROI), which refers to a specific region of the image data defined and selected for a specific analysis or purpose. Areas of the bone with mineral deposits were manually sectioned out or removed by thresholding so that they would not interfere with bone microarchitecture output. Edited scans were then analyzed using the Bone Microarchitecture Analysis (BMA) app within the software to assess the standard American Society for Bone and Mineral Research (ASBMR) microarchitecture measures [58]. A calibration phantom was utilized to calibrate the scans to generate TMD scores. As the calculation for trabecular BMD (TMD) in the Analyze software algorithm is designed for use in living specimens rather than archaeological samples in which there is a degree of diagenesis, the calibrated scores for each individual may not reflect the true mineral density scores in mg HA (hydroxyapatite)/cm^3^ for comparison with fresh bone. Though previous archaeological studies have used methods such as DEXA to assess archaeological remains and, in some cases, compare them to modern populations, others have found that the diagenesis of archaeological specimens precludes them from being directly compared to modern clinical scores and have found low correlations between X-ray density measurements and morphological analysis of trabecular variables such as bone volume fraction [59]. Thus, the generated TMD values were converted into standardized z-scores for each sample separately to use for comparative purposes within the Tombos individuals only.

## Data Availability

The authors confirm that the data supporting the findings of this study are available within the article. Access to the archaeological skeletal remains can be requested by contacting the curator, Dr. Michele Buzon, at Purdue University.
